# Investigating the Plasmon Resonances of Silicon Nanowires without Oxide Shell

**DOI:** 10.1021/acsomega.6c02153

**Published:** 2026-06-13

**Authors:** Rizwan Rafique, Antonino La Magna, Antonio Massimiliano Mio, Salvatore Patanè, Jost Adam, Rosaria Anna Puglisi

**Affiliations:** † CNR—Istituto per la Microelettronica e Microsistemi, Strada Ottava 5, Zona Industriale, Catania 95121, Italy; ‡ Department of Mathematics and Computer Science, Physics and Earth Science (MIFT), University of Messina, Viale F. Stagno d’Alcontres 31, Messina 98166, Italy; § Computational Materials and Photonics, FB 16—EECS and FB 10—Physics, 9178 University of Kassel, Wilhelmshöher Allee 71, Kassel D-34121, Germany; ∥ Center for Interdisciplinary Nanostructure Science and Technology (CINSaT), University of Kassel, Heinrich-Plett-Str. 40, Kassel D-34132, Germany

## Abstract

Silicon nanowires have attracted the scientific community’s attention due to their exceptional optical properties and potential for device integration. In our previous research, we investigated plasmonic resonance behavior in silicon nanowires to elucidate the physical origin of plasmonic excitations in semiconductor nanostructures and to explore plasmonic responses for potential applications in silicon-based nanophotonic and optoelectronic devices. We directly observed the longitudinal plasmonic resonance and the transverse plasmonic resonance in cylindrical and conical silicon nanowires with diameters of 30–100 nm. In our most recent work, we extended the study to conical-shaped silicon nanowires with quantistic NW tip sizes to further understand the morphological influence on the plasmonic resonances. All our previous findings refer to silicon nanowires enveloped in a silicon oxide shell. It is known that the dielectric shell modulates the local electromagnetic field and resonance conditions. In this paper, we present our investigation of several groups of conical and cylindrical silicon nanowires with varying lengths, without a dielectric shell, suspended in vacuum or deposited on a carbon film. We employed a scanning transmission electron microscope equipped with electron energy-loss spectroscopy. The results indicate that all the investigated SiNW groups exhibit signals with a periodic nature, with a clear, strong dependence of the plasmonic resonance energy on NW length rather than on environmental conditions, and confirm a strong transversal plasmonic resonance signal in all cases. This opens a practical pathway to plasmon engineering in silicon nanowires solely through geometry, simplifying their integration into nanophotonic platforms.

## Graphical abstract

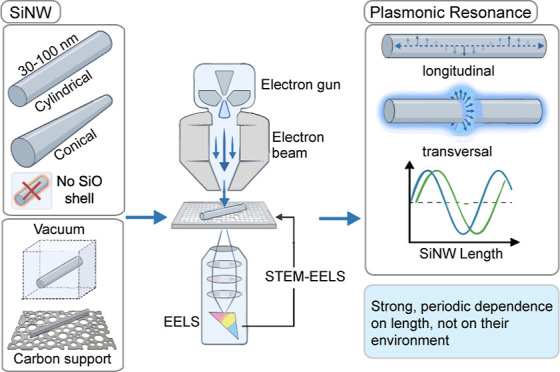

## Introduction

Nanowires (NWs) have garnered considerable attention within the scientific community due to their exceptional optical properties and VLSI integration capabilities. Recent research efforts have focused particularly on silicon NWs due to their abundance, stability, and nontoxicity.^1,2^ A plasmonic resonance (PR) occurs when the incident radiation’s frequency aligns with the natural oscillation frequency of the electrons, resulting in a localized enhancement of the electromagnetic field.^3^ PR is widely studied in the photonics and electronics communities and exploited because it enhances the local electric field by concentrating electromagnetic radiation into subwavelength volumes.^4–8^ In the literature, metallic nanostructures have been widely studied, but only recently have a few reports appeared on PR in silicon nanowires (SiNWs).^9–13^ The reason is that studying PR in semiconductor nanostructures is quite challenging. In our recent studies, we have successfully demonstrated the direct observation of PR in SiNWs by scanning transmission electron microscope (STEM) analysis coupled with in situ scanning transmission electron microscope (EELS), proving it to be one of the most effective techniques for studying these plasmonic phenomena with high spatial and energy resolution.^14,15^ We demonstrated PR on differently shaped—cylindrical and conical—SiNWs.^16,17^ Specifically, we focused on cylindrical-shaped SiNWs and reported the appearance of both plasmonic resonance (LPR) and transversal plasmonic resonance (TPR) in these structures within the UV region of the spectrum.^15,16^ In analogy to the metallic counterparts,^18^ we identified several harmonic modes of the PR that exhibit characteristics peculiar to metallic nanomaterials, such as damping, periodicity, and spatial distribution. The signals, however, unlike in metallic materials, appeared elongated in space and persisted over a wide energy range, resulting in broad peaks, as expected for semiconductor nanostructures.^16^ In another study, we examined tapered SiNWs and observed TPR with continuous signals extending along their length.^19^ In our most recent work, we analyzed the PR behavior of conical-shaped SiNWs with tip diameters in the quantum regime and compared these results with those for cylindrical SiNWs.^17^ The findings indicated the presence of LPR, pointing to the role of quantum size in the plasmon resonance. All previous studies have used SiNWs embedded in a silicon oxide shell and suspended in vacuum. In metal–dielectric systems, the surrounding dielectric plays a role in defining the conditions under which plasmonic resonances occur, because it generates an induced field that partially counteracts the electron restoring force, typically leading to a redshift of the resonance as the environmental permittivity increases.^20,21^ For the reasons above, it is important to understand whether removing the dielectric shell in semiconductor nanostructures could, as in their metallic counterparts, modify the resonance conditions, shift the modal energies, and alter the field distribution. This consideration provides the rationale for investigating shell-free nanowires and for directly comparing their plasmonic response with that of oxide-coated counterparts. In the present paper, we investigated three groups of shell-free SiNWs with conical shape and two groups with cylindrical shape, with different lengths and diameters, suspended in vacuum or lying on a carbon film. In addition, plasmonic resonances were also investigated in oxide-coated cylindrical nanowires, enabling a direct comparison between structures with and without the dielectric shell. The groups of conical SiNWs have lengths ranging from 180 to 340 nm. The groups of cylindrical ones range between roughly 230 and 330 nm. The selection of these shell-free groups is motivated by the need to systematically investigate the influence of dielectric shells on PR in different surrounding environments (suspended in vacuum versus lying on a carbon film). To complete the study, we also analyzed a core–shell SiNW, with a length of 230 nm, a diameter of 23 nm, and an oxide thickness of 4.5–5 nm, and directly compared the results with our recently reported core–shell SiNWs. These nanostructures were synthesized by using the liquid–solid–vapor growth method.^22–35^ After the SiNWs deposition, we removed the gold catalysts by using a method described elsewhere,^36^ and no detectable Au-related signal was observed by EDX analyses performed on each investigated nanowire. Prior to TEM analysis, the samples were treated with a diluted HF solution in order to remove the native oxide layer from the SiNW surface. Immediately after the etching step, the samples were transferred into the TEM holder and inserted into the microscope, thereby minimizing exposure to ambient conditions and limiting the possibility of reoxidation. No measurable oxide layer was detected after HF treatment. The measurements were performed using a Cs-corrected JEOL ARM 200F S/TEM equipped with a cold-field emission gun and a monochromator, providing sub-Ångström spatial resolution and an energy resolution of 0.3 eV. Under these conditions, oxide layers with thicknesses of 1 nm or less can be reliably identified. Depending on the sample group, between 10 and 15 individual nanowires were analyzed. The results indicate that all investigated SiNW groups exhibit features similar to those previously observed, showing a harmonic response despite the absence of a dielectric shell.^15,16,18^ Our work aims to elucidate the role of the dielectric shell in shaping the plasmonic response of silicon nanowires by providing a systematic experimental investigation and a numerical study of shell-free conical and cylindrical SiNWs. By removing the oxide shell and analyzing nanowires with different geometries, lengths, and environmental configurations, we directly assess if and how dielectric screening and boundary conditions affect the excitation, energy, and spatial distribution of longitudinal and transverse plasmon resonances. This approach enables a direct comparison with previously studied core–shell SiNWs and provides deeper insight into the intrinsic plasmonic behavior of silicon nanostructures.

## Results and Discussion

Table 1 reports the geometric parameters of all the shell-free and core–shell conical and cylindrical SiNWs investigated in this work.

**Table 1:** Geometrical Parameters of all Groups of Shell-Free/Core–Shell SiNWs Reported in this Work

SiNWs shape	length (nm)	diameter at base (nm)	diameter at center (nm)	diameter at tip (nm)
conical	180	37	30	25
conical	280	40	30	15
conical	340	40	35	22
cylindrical	270	45	-	-
cylindrical	325	50	-	-
cylindrical core–shell	230	23	-	-

Figure 1 shows the characterization results of the first set of shell-free conical SiNWs. Panels (b–c) display the energy-filtered spectroscopic images (EFSIs) acquired at two distinct energy values. The rectangular insets indicate the regions for the signal extraction reported in Figure 1d. The curves indicate that at 4.0 eV, a single peak emerges at 110 nm from the SiNW base (red curve). This feature is plausibly attributed to an interband transition^37,38^ or to scattering effects.^15^ At 5.5 eV, two distinct peaks appear, as indicated by the arrows: one located near the base and the other near the tip. The two peaks can be ascribed to localized plasmon resonance (LPR) propagating within a one-dimensionally confined harmonic resonator, where the standing-wave condition gives rise to well-defined nodes and antinodes along the structure, corresponding to minima and maxima of the electromagnetic field distribution. In general, the signals appear spatially extended, giving rise to broader peaks compared to those typically observed in metallic resonators, in agreement with previous observations and expectations for semiconductor nanostructures.^16^ The peak observed at the SiNW base can be attributed to the well-known point effect, widely reported in the literature,^16^ which induces field enhancement at the extremities of the structure, acting as the oscillator’s nodal points. The data observed so far are in strong agreement with those previously reported for core–shell nanowires, 189 nm-long.^15^ From these data, we preliminarily conclude that the resonator length is an important parameter for observing longitudinal modes, independent of the dielectric shell. The successive result sections will further discuss this hypothesis.

**Figure 1:**
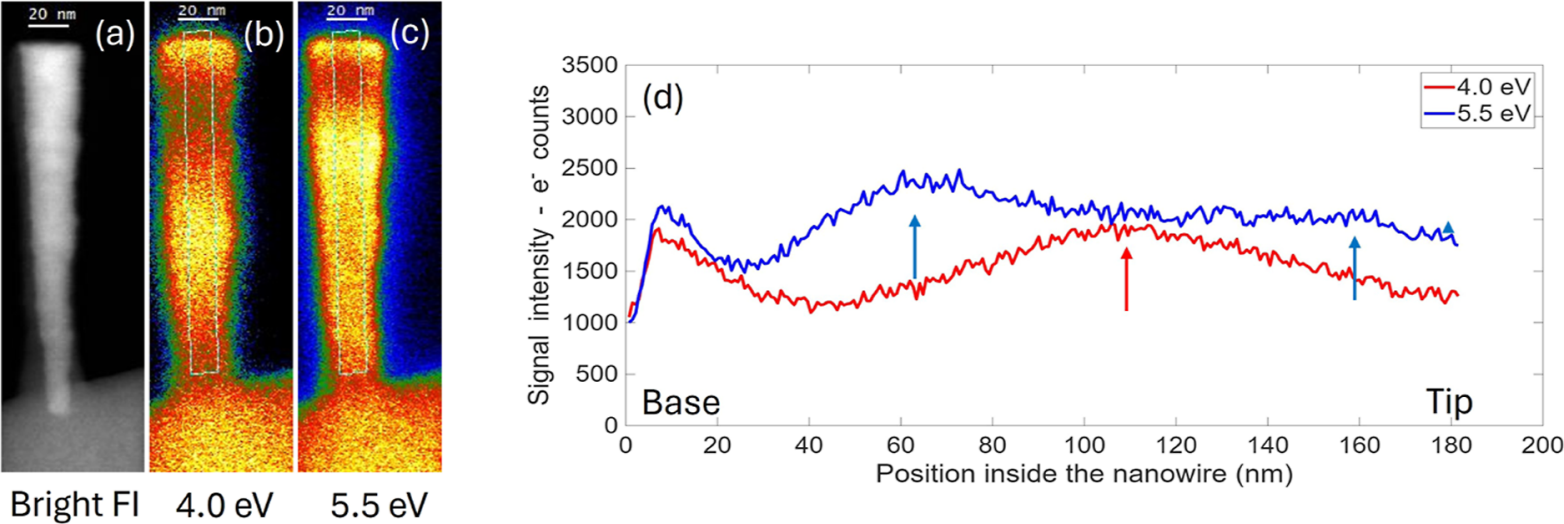
(a) Bright field image of the 180 nm long shell-free SiNW, attached to the base of the TEM grid (from the tip). (b,c) EFSI maps of SiNWs collected at two different energy values. (d) Signal intensity extracted along the NW axis from the white rectangles imaged in (b) to (c), and plotted as a function of the position inside the NW going from top to bottom. The arrows indicate the position of the resonance lobes.

Figure 2a–f) presents, on the left, the EFSI maps of the same nanowire acquired at higher energies, indicating a decrease of the signal intensity at the SiNW center, and an increment along the SiNW sidewalls. Figure 2g reports the signals extracted from the white rectangular regions, showing that the signal is higher in correspondence of the SiNW walls with respect to the center. The intensity increases from 6.5 eV up to 8.5 eV. In comparison, at 9.0 eV, the intensity begins to decrease.

**Figure 2:**
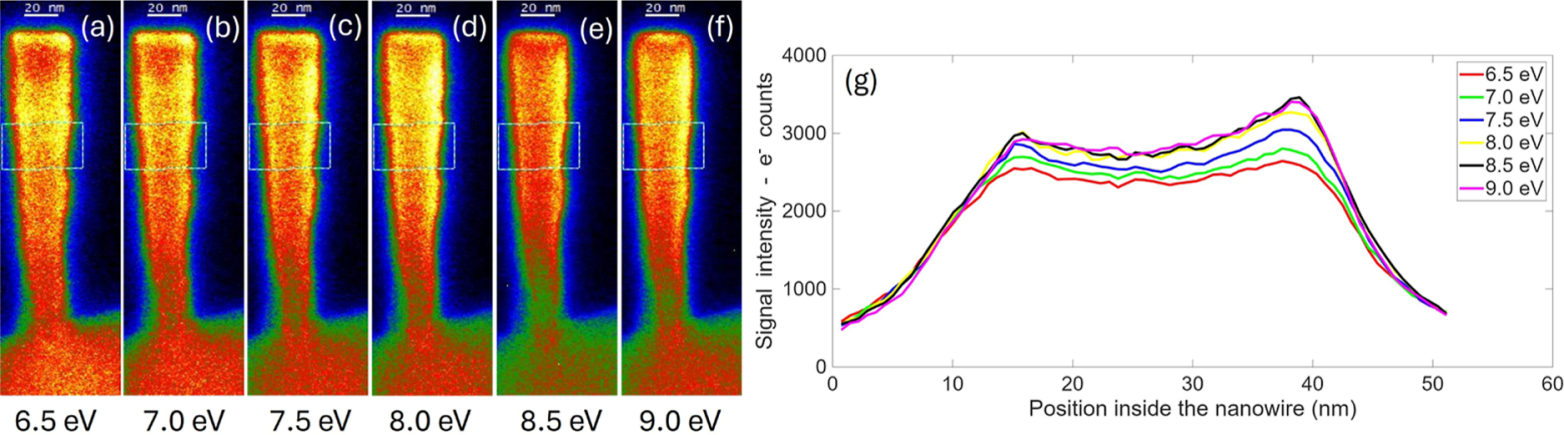
(a–f) EFSI maps of the SiNWs pictured in Figure 1, extracted at energy between 6.5 and 9 eV. (g) Signal intensity as a function of position, extracted along the diameter, in correspondence with the white rectangles imaged in (a–f).

Figures 3, 4, and 5 present the characterization results of the second set of shell-free conical SiNWs. This group is suspended in a vacuum and attached to the TEM grid via the nanowire base. As in the previous case, and hereafter, all white rectangular insets within the EFSI maps denote the regions used for signal extraction.

**Figure 3:**
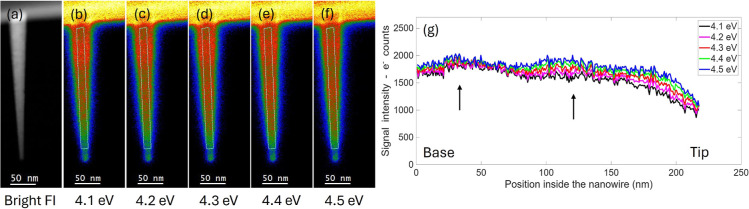
(a) Bright field image of one of the 280 nm long shell-free SiNWs attached to the TEM grid from the base. (b–f) EFSI maps of SiNWs extracted at five different energy values. (g) Signal intensity extracted along the NW axis from the white rectangles imaged in (b) to (f), and plotted as a function of the position inside the NW, going from top to bottom. The arrows indicate the position of the resonance lobes.

**Figure 4:**
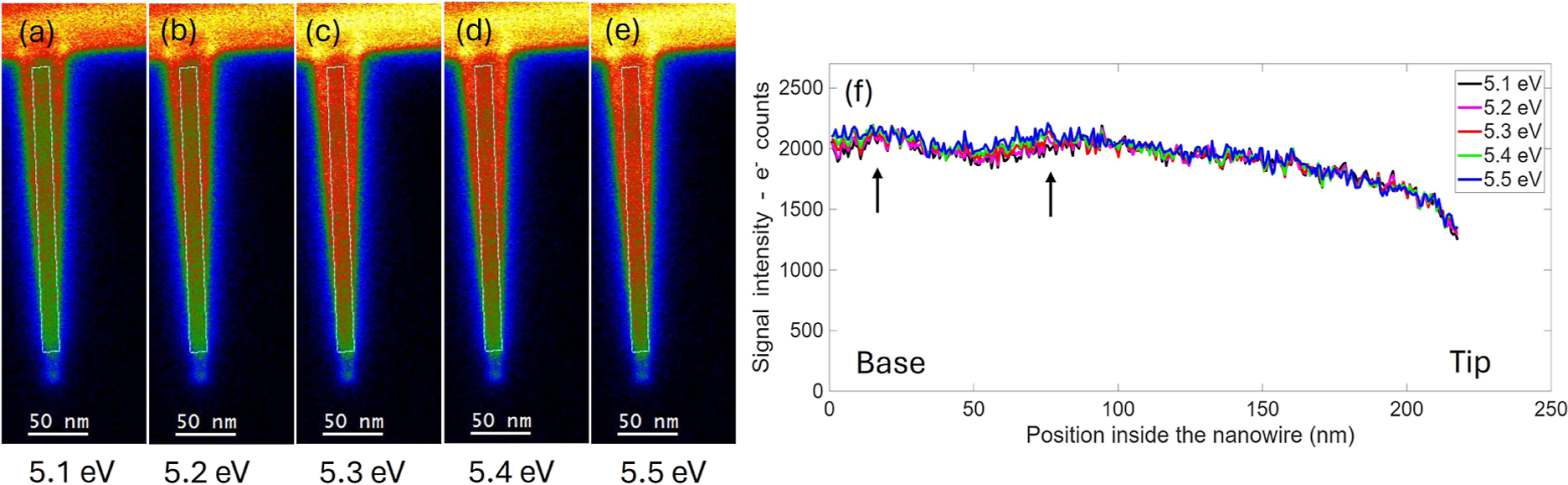
(a–e) EFSI maps for the NW imaged in Figure 3 (a), extracted at higher energy values. (f) Signal intensity as a function of position for several energies. The signal-intensity extraction is analogous to that in Figure 3g. The arrows indicate the position of the resonance lobes.

**Figure 5:**
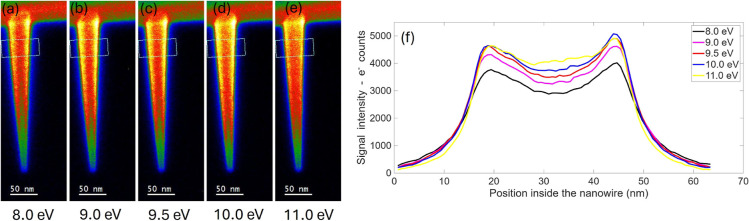
(a–e) EFSI maps of SiNWs imaged in the range 8–11 eV. (f) Signal intensity as a function of position, extracted along the diameter, in correspondence with the white rectangles imaged in (a–e).

Figure 3b–f presents the EFSI maps obtained at different energies. Figure 3g illustrates the spatial evolution of the signal intensity along the nanowire, revealing two pronounced maxima: one located near the base and the other at the center of the SiNW. This spatial distribution is consistent with the excitation of the fundamental LPR.

Figure 4a–e presents the EFSI maps of the same family of SiNWs acquired over the energy range from 5.1 to 5.5 eV. Figure 4f reports the signal intensity profiles extracted along the nanowire longitudinal axis at the different investigated energies. Both the EFSI maps and the corresponding intensity profiles reveal only two pronounced peaks. Compared to the behavior observed at lower energies around 4.1–4.5 eV (Figure 3g), the signal positions shift clearly toward the SiNW base. Specifically, the first peak appears at approximately 15 nm from the base and the second at about 75 nm, whereas in the 4.1–4.5 eV range the corresponding peaks were located at 35 and 120 nm, respectively. These systematic peak position shifts toward the base at higher energies suggest the excitation of a second-order harmonic mode. Under this condition, one might expect the emergence of a third resonance peak near the nanowire tip. However, at 5.5 eV, the EFSI maps show no evidence of a third localized spot. The clear visibility of the first two plasmonic lobes, accompanied by the strong suppression of the third one, can be rationalized by considering size-confinement effects. As the nanowire diameter approaches 15 nm, electron motion becomes increasingly restricted, leading to the damping of higher-order modes and, consequently, to the absence of the third lobe.^15^ Overall, these observations provide strong evidence for the excitation of the second harmonic of the localized plasmon resonance.

Figure 5a–e presents, on the left, the EFSI maps, which clearly reveal signals distributed along the SiNW sidewalls. Figure 5f highlights the TPR signals increasing in intensity from 8.0 eV and reaching a maximum at 10.0 eV. At 11.0 eV, indeed, the TPR intensity decreases. Figures 6, 7, and 8 present the characterization results on the third group of shell-free conical SiNWs. They are not attached to the base and are lying on the carbon (C) film of the TEM grid.

**Figure 6:**
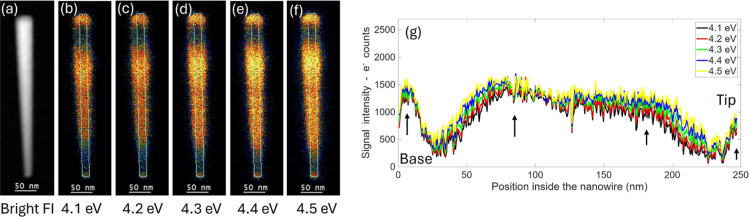
One of the SiNWs from the group of 340 nm long shell-free SiNW, lying on the C film: (a) bright field image, (b–f) EFSI maps, and (g) signal intensity extracted as in the previous cases, and plotted as a function of the position going from top to bottom. The arrows indicate the position of the resonance lobes and at the NW ends.

**Figure 7:**
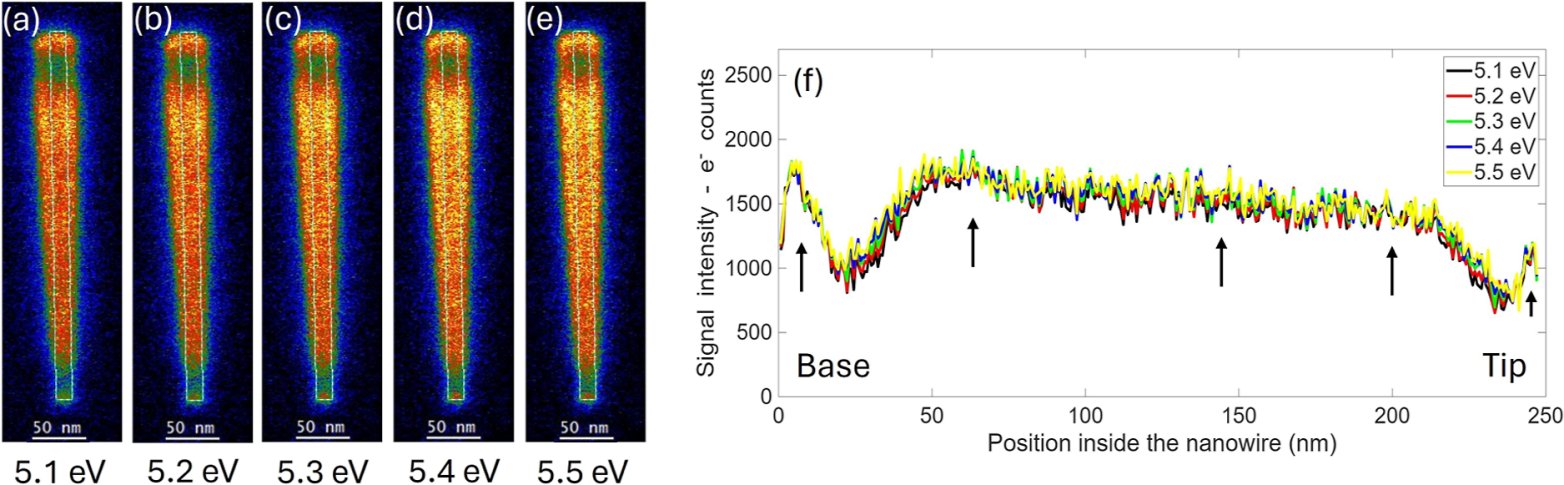
(a–e) EFSI maps for the NW imaged in Figure 6 (a), signal intensity as a function of position (f) extracted as in Figure 6g. The arrows indicate the position of the resonance lobes and the signals at the ends.

**Figure 8:**
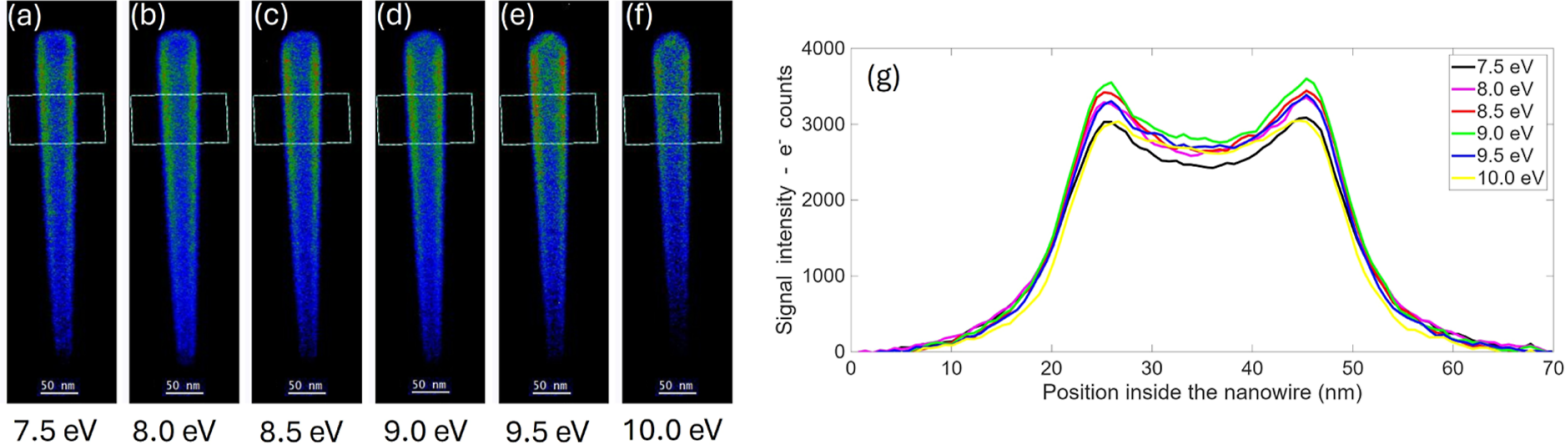
(a–f) EFSI maps for the NW imaged in Figure 6a extracted in the energy range of the TPR resonances. (g) Signal intensity as a function of position, extracted along the diameter, in correspondence of the white rectangles imaged in (a–f).

Figure 6b–f presents the EFSI maps (left) and the corresponding extracted intensity profiles (right) acquired in the 4.1–4.5 eV energy range. The graph reveals four distinct peaks, as indicated by the arrows. The two peaks located at the extremities of the SiNW can be attributed to a tip effect, as discussed previously. In contrast, the two central peaks indicate the excitation of the fundamental localized plasmon resonance mode. Despite the absence of a dielectric shell and the fact that this group of nanowires is onto a carbon film rather than being suspended in vacuum, the energy of the fundamental mode remains unchanged and coincides with that measured for the core–shell structures.

Figure 7a–e presents the EFSI maps and the corresponding extracted intensity profiles for the same family of SiNWs shown in Figure 6, acquired in the 5.1–5.5 eV energy range. In this case, apart from the two peaks at the extremities of the nanostructure, whose presence has already been clarified, the energy distribution along the nanowire is dominated by three peaks located in the central region. Therefore, as expected, increasing the energy leads to the excitation of the second harmonic of the localized plasmon resonance. In particular, the absence of the dielectric shell does not appear to play a significant role in this case, since the emergence of the second harmonic is observed at energies similar to those measured for the core–shell structures (5–5.5 eV).^15^

As the energy increases, the EFSI maps highlight the presence of the TPR (Figure 8a–f), whose intensity increases to a maximum at 9.0 eV (Figure 8g) and then decreases at higher energies.

In the following section, we present the results obtained for the shell-free cylindrical SiNW groups. TEM analyses were performed only on a portion of each nanowire, as the remaining segments were in direct contact with either the Si substrate or the carbon grid. Including the full SiNW geometry would introduce signal attenuation effects, thereby hindering the extraction and interpretation of the plasmonic resonance response.

Figures 9 and 10 present the characterization results for the fourth group of shell-free SiNWs with cylindrical geometry, aimed at elucidating the role of morphology in shell-free structures and enabling a direct comparison with previously reported findings. Figure 9b–d reports the EFSI maps acquired at three distinct energy values. In the 4.7 eV energy range, a single lobe appears (Figure 9e). By symmetry, we expect a second lobe on the opposite SiNW side, and this behavior is consistent with the excitation of the fundamental localized plasmon resonance (LPR) mode. At higher energies, at 5.7 eV, a one-and-a-half-lobe pattern emerges, with the remaining half-lobe presumably located on the opposite side of the nanowire, again dictated by symmetry considerations, suggesting the excitation of the second harmonic of the LPR. As in previous cases, we attribute the signals detected at the nanowire tips to tip-induced field enhancement. The 3.6 eV, as in the case above, is presumably related to scattering or an interband transition.

**Figure 9:**
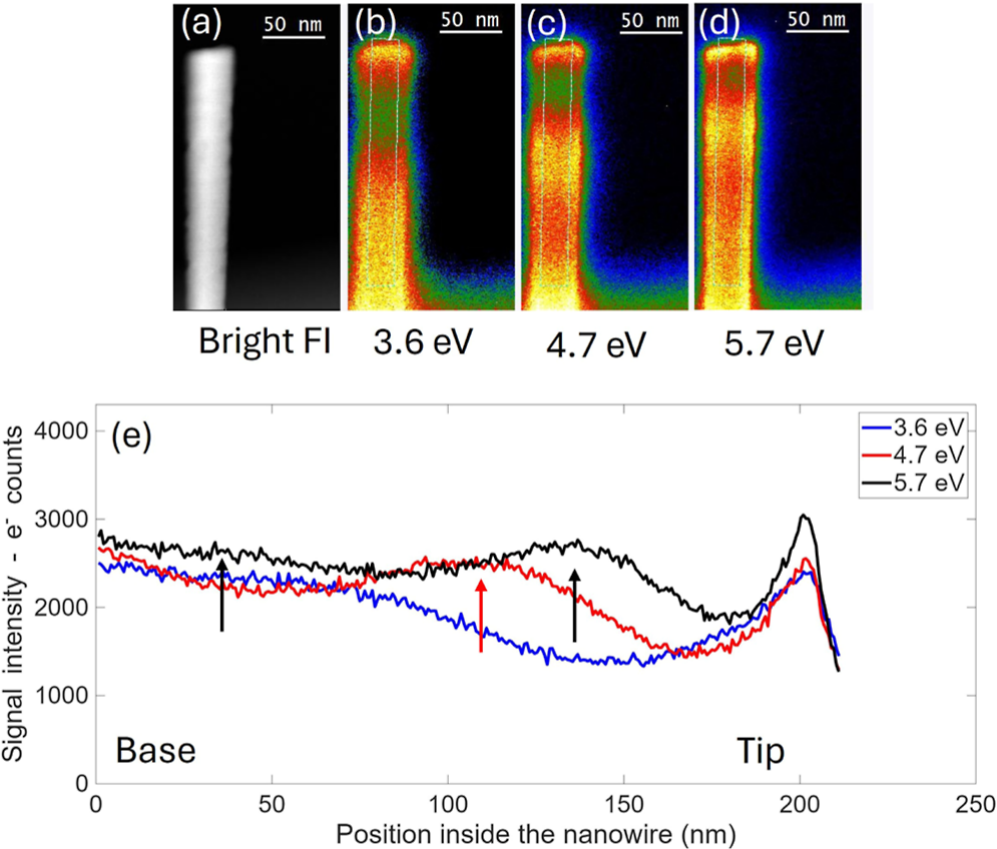
SiNW from the group of the 270 nm long shell-free cylindrical structures: (a) bright field image, (b–d) EFSI maps, (e) signal intensity extracted along the NW axis from the white rectangles in (b–d), and plotted as a function of the position inside the NW. The arrows indicate the position of the resonance lobes.

**Figure 10:**
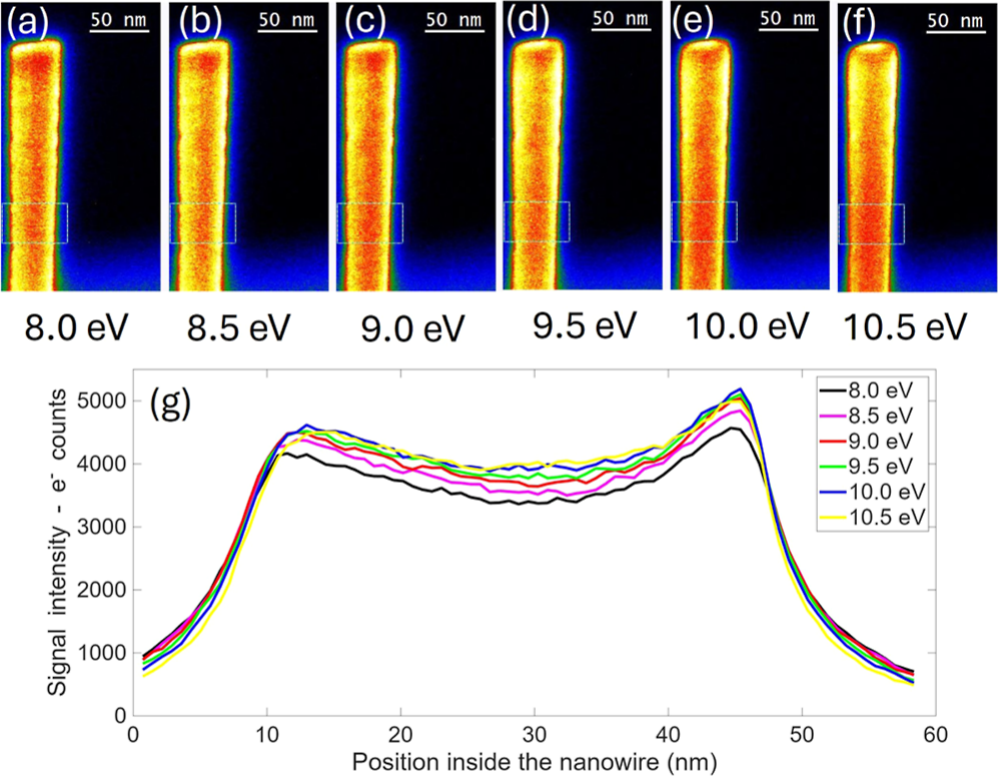
(a–f) EFSI maps for the NW imaged in Figure 9a,g signal intensity extracted along the diameter, in correspondence of the white rectangles imaged in (a–f).

Figure 10a–f displays the EFSI maps, revealing a progressive relocation of the signal along the SiNW sidewalls with increasing energy, consistent with the excitation of the TPR. As shown in Figure 10g, the TPR emerges at 8.0 eV, reaches maximum intensity at 10.0 eV, and subsequently decreases at higher energies (10.5 eV), identifying 10.0 eV as the peak TPR response.

Figures 11 and 12 present the characterization results of the fifth group of shell-free cylindrical SiNWs. As shown in Figure 11, signal localization at the SiNW tips is observed, consistent with the tip effect previously discussed.

**Figure 11:**
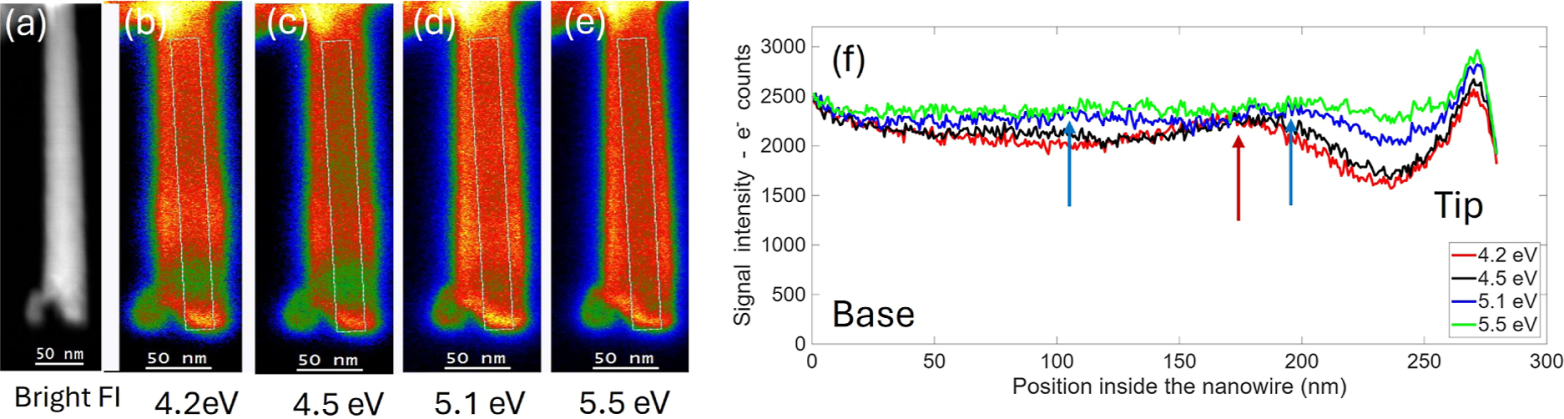
(a) Bright field image of a 325 nm long shell-free cylindrical SiNW. (b–e) EFSI maps extracted in the range 4.2–5.5 eV, (f) signal intensity extracted as in the previous cases.

**Figure 12:**
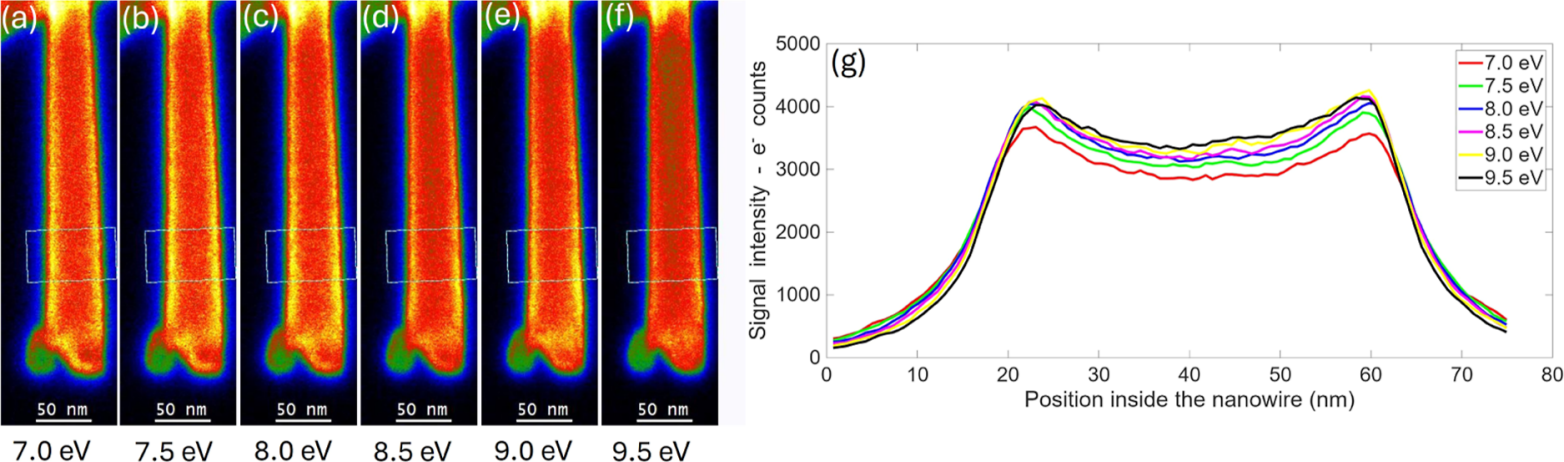
(a–f) EFSI maps relative to the NW imaged in Figure 11a extracted in the TPR energy range, and corresponding signal intensity­(g).

Figure 11b–e shows the EFSI maps acquired at different energy values, while Figure 11f reports the corresponding extracted signals. A first lobe appears at 4.2 eV (red arrow), and a second lobe is likely present on the opposite side of the SiNW, indicating excitation of the fundamental LPR. At 5.1 eV, one and a half lobes are observed (blue arrows), with the remaining portion presumably located on the opposite side of the nanowire, consistent with excitation of the LPR second harmonic. The measurements performed on a 230 nm long core–shell SiNW (not shown here) from the sixth group of nanostructures examined in this paper exhibit the first and second harmonics at 5.3 and 6.0 eV, respectively.

As in the previous cases, the Figure 12a–f presents EFSI maps showing a progressive shift of the signals toward the sidewalls of the SiNWs, confirming the presence of TPR in these structures. In particular, as shown in Figure 12g, the TPR is already observable at 7.0 eV, and its intensity increases with increasing energy, reaching a maximum at 9.0 eV. At higher energies, the signal intensity decreases, suggesting the progressive disappearance of plasmonic features.

Overall, the experimental observations demonstrate that plasmonic resonances in silicon nanowires are robust features of the nanostructure geometry and electronic confinement, persisting even in the absence of a dielectric shell. Both the fundamental and the second longitudinal plasmon resonance remain largely unaffected in SiNWs, indicating that dielectric screening and geometry do not influence mode confinement or resonance conditions. At the same time, as demonstrated before, the nanostructure length plays a fundamental role, while the presence of an oxide shell has little to no effect on the transverse plasmon resonances, whose spectral and spatial characteristics are primarily determined by the intrinsic electromagnetic response of the silicon nanowires and their length rather than by the oxide shell.

## Numerical Methods

We performed density-functional theory (DFT) calculations to calculate the optical dispersion data for the shell-free conical SiNWs, following our previous paper.^16^ We performed finite-element method (FEM) simulations to model the optical response of shell-free conical SiNWs. We used the wave optics module of the commercially available COMSOL Multiphysics FEM solver.^39^ We excited the structure with a background electromagnetic plane wave (wavelengths between 170 and 420 nm, corresponding to energies between about 7 and 3 eV, respectively).

This plane wave is impinging perpendicularly to the main SiNW axis $\left(\mathrm{k⃗}=k_{x}\hat{\vec{x}}\right)$ , where the electrical field is either polarized along this axis (*y*—direction) for the (predominant) optical excitation of longitudinal plasmons, or perpendicular to this axis, predominantly exciting transverse plasmons.

### Numerical Results and Comparison with the Experimental Characterization

To explain the physics behind our experimental results, we calculated the optical absorption and scattering cross sections, along with the corresponding electromagnetic (EM) near-field patterns (Figures 13 and 14, respectively). The simulated shell-free conical SiNW is 340 nm long. It is important to note that the background electrical field is impinging on the SiNW surface from the positive *x*-direction, leading to a single-sided resonance, contrary to the experimental results showing resonances with rotational symmetry around the longitudinal nanowire (*y*-) axis. Figure 13 reports optical dispersion data for the shell-free conical SiNW. Figure [Fig fig1]3 (a) shows the longitudinal polarization, and Figure [Fig fig1]3 (b) the perpendicular polarization with respect to the NW’s long axis. In longitudinal polarization, the SiNW begins absorbing the EM field at approximately 2.5 eV. The absorption increases up to 2.9 eV, after which it plateaus. A further increase in energy produces a sharp peak at around 4.0 eV, which corresponds to one of the prominent plasmon peaks and some scattering signals in the SiNW. For energy values above 4 eV, several small peaks appear, indicating higher-order resonances. The scattering curve closely follows the absorption spectrum, except at 2.5 eV. The transverse polarization, Figure [Fig fig1]3 (b), shows a significant signal at the energy of 11 eV. The electromagnetic field interacts with the SiNW, resulting in a higher scattering rate than absorption in this energy range. The simulated spectrum exhibits broad peaks at high energies for transverse polarization, where in the other regime, the peaks disappear. This trend indicates the transition from longitudinal to transverse resonance observed experimentally.

**Figure 13:**
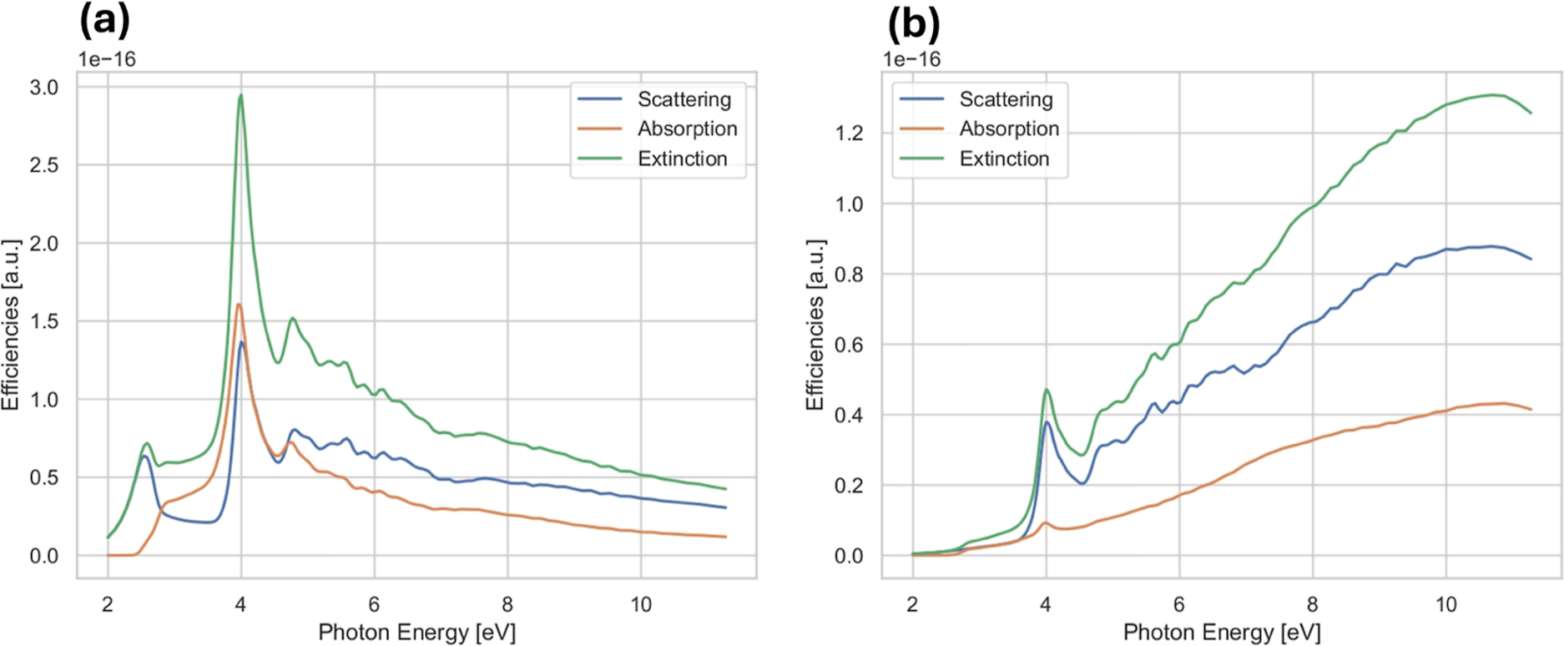
Optical response (scattering, absorption, and extinction efficiencies) of a simulated 340 nm long shell-free conical SiNW. (a) Longitudinal polarization, and (b) transverse polarization (with respect to the NW’s long axis).

**Figure 14:**
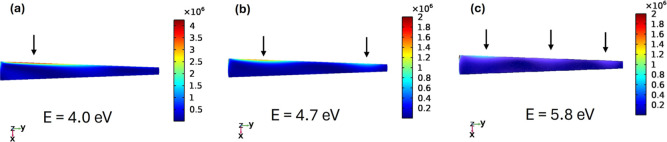
Electromagnetic power loss (PL) [W/m^3^] on the simulated shell-free conical SiNW with a length of 340 nm, at 4.0 eV (a), 4.7 eV (b), 5.8 eV (c). The excitation is optical, with an incident electromagnetic plane wave coming from the top (positive *x* direction).

To validate our results, we extracted the simulated electromagnetic power loss (PL) profiles for the plasmon modes of a shell-free conical SiNW with a length of 340 nm, as shown in Figure 14. Figure 14a shows the appearance of one spot indicated by the black arrow at an energy value of 4.0 eV. Figure 14b shows the appearance of two spots indicated by the two black arrows—at an energy value of 4.7 eV, probably due to plasmons, specifically the LPR. In comparison, the experimentally obtained TEM results show two peaks in the 4.5 eV energy range. Figure 14c shows the appearance of three spots indicated by the three black arrows along the SiNW length at an energy of 5.8 eV, which confirms the presence of multiple LPR harmonics. From the experimental results, we observed three peaks at energies between 5.5 eV, i.e., again at similar energies to the simulated peaks. It is important to note that the simulation results show the signals on one side of the SiNWs, as the structure was excited by a background electromagnetic plane wave in a single direction. The FEM simulations were performed under plane-wave excitation, thereby providing direct access to the nanowires’ optical (radiative) response. The excellent agreement between simulated optical resonances and experimentally observed EELS features indicates that the detected modes include optically active plasmonic excitations.

## Summary and Conclusions

To summarize, we report in Figure 15 the data from all the shell-free conical and cylindrical nanostructures studied in this work, together with the results from literature on core–shell SiNWs,^16^ as a function of the NW length. The structures from the literature have a cylindrical shape and an oxide shell with 4.5–5 nm thickness, and the LPR appears as follows, approximately a similar trend for the energy values obtained here,^15–17^ independently of the NW shape and regardless of whether they are attached to the base, suspended in the vacuum or lying on a carbon film.

**Figure 15:**
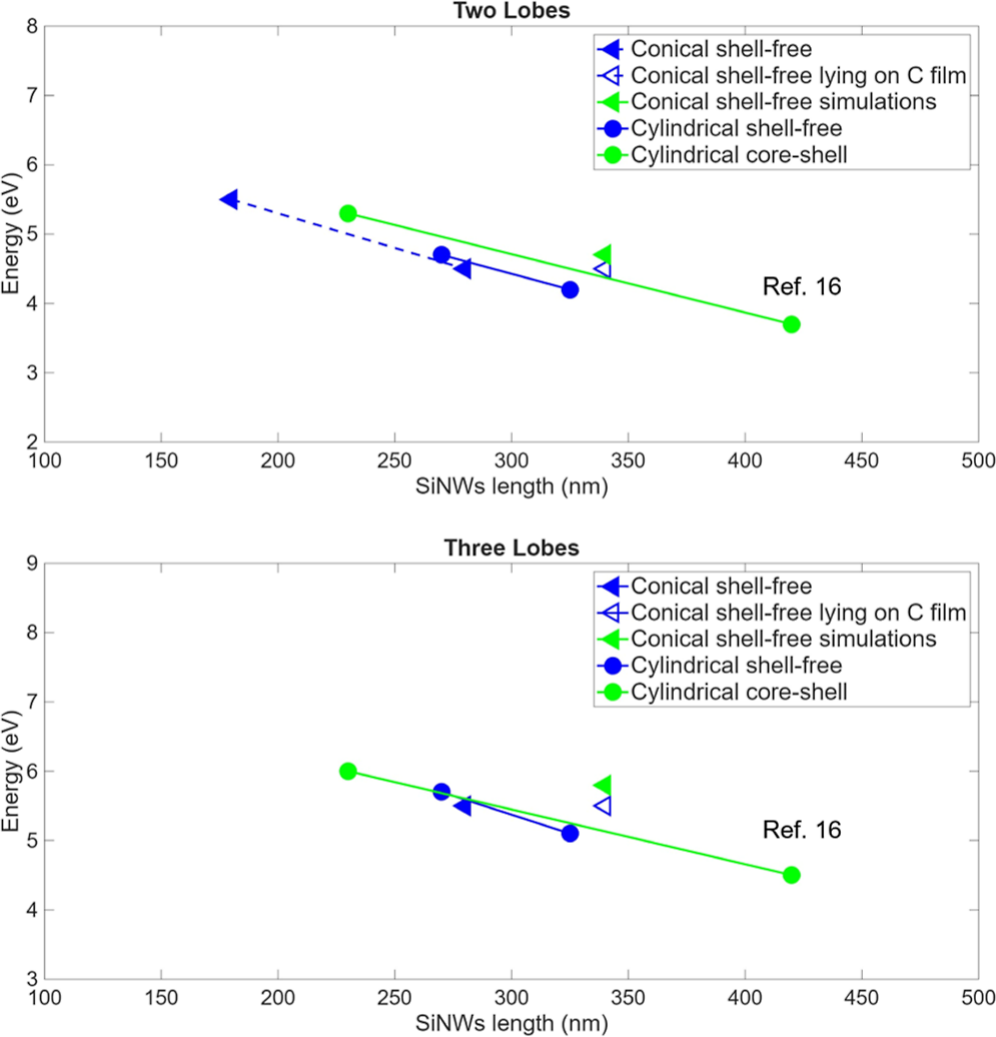
Comparison of experimental and simulation data, for the fundamental (a) and second harmonic (b) plasmonic resonance of core–shell and shell-free SiNWs with different shapes.

Note that, in the case of shell-free conical SiNWs, the second harmonic of LPR appeared in the groups of SiNWs with a total length larger than 280 nm. The LPR second harmonic absence in the shell-free conical SiNWs 180 nm long could be related to the smaller length of these nanostructures. This effect is in agreement with the previous findings in literature.^15^ Indeed, in prior studies, we observed more harmonic modes in cylindrical SiNWs with a length of 400 nm, whereas only the fundamental LPR surfaced in 200 nm-long structures.^15^ These results are consistent with those observed here.

Furthermore, the simulation results support the experimental evidence with a slight difference in energy values as reported in.^16^

In summary, the results of all investigated groups of conical shell-free and cylindrical shell-free SiNWs suggest the presence of LPR and confirm the clear appearance of TPR signals. The signals appear regardless of whether the SiNW is suspended in a vacuum or lying over the carbon film. In all the studied cases, we observed that the LPR appeared within the energy ranges 4.0–5.5 eV for the first mode and 4.5–6.0 eV for the second mode. Across all conical shell-free SiNWs, the first and second harmonic PRs appear approximately at similar energy values reported previously in the core–shell ones, depending only on the NW length, as demonstrated also in previous studies on core–shell NWs. The longer SiNWs exhibited the second resonance mode, consistent with our previous findings on cylindrical core–shell SiNWs, which were also confirmed by simulations. The optical response and corresponding EM near-field patterns of the conical shell-free SiNWs strongly support the experimental results.

In conclusion, this study establishes that silicon nanowires intrinsically support both longitudinal and transverse plasmon resonances, whose existence does not depend on a dielectric shell. The comparative analysis of shell-free and core–shell nanowires reveals that dielectric screening does not affect the energy or visibility of the plasmonic modes, leaving the resonance features largely unchanged across all the investigated geometries. Spanning several shapes, spatial arrangements, and dielectric configurations, the most important parameter affecting the resonance energy is the NW length. This finding clearly indicates that the phenomenon can be modulated and controlled reliably by tuning the nanostructure length. Furthermore, our study clarifies the physical origin of plasmonic excitations in semiconductor nanowires. It provides essential guidelines for tailoring plasmonic responses in silicon-based nanophotonic and optoelectronic devices through geometry and dielectric engineering rather than solely material composition.

## References

[1] Kayes B. M., Atwater H. A., Lewis N. S. (2005). Comparison of the device physics
principles of planar and radial p–n junction nanorod solar
cells. J. Appl. Phys..

[2] Stelzner T., Pietsch M., Andrä G., Falk F., Ose E., Christiansen S. (2008). Silicon nanowire-based
solar cells. Nanotechnology.

[3] Maier, S. A. Plasmonics: Fundamentals and Applications; Springer, 2007.

[4] Shao M., Cheng L., Zhang X., Ma D. D. D., Lee S.-T. (2009). Excellent
photocatalysis of HF-treated silicon nanowires. J. Am. Chem. Soc..

[5] McAlpine M. C., Ahmad H., Wang D., Heath J. R. (2007). Highly ordered nanowire
arrays on plastic substrates for ultrasensitive flexible chemical
sensors. Nat. Mater..

[6] Pandey R. R., Alshahrani H. S., Krylyuk S., Williams E. H., Davydov A. V., Chusuei C. C. (2018). Electrochemical detection of acetaminophen with silicon
nanowires. Electroanalysis.

[7] Moeinian A., Gür F. N., Gonzalez-Torres J., Zhou L., Murugesan V. D., Dashtestani A. D., Guo H., Schmidt T. L., Strehle S. (2019). Highly localized
SERS measurements using single silicon nanowires decorated with DNA
origami-based SERS probe. Nano Lett..

[8] Banerjee D., Guo X., Cloutier S. G. (2018). Plasmon-enhanced
silicon nanowire array-based hybrid
heterojunction solar cells. Sol. RRL.

[9] Losquin A., Kociak M. (2015). Link between cathodoluminescence and electron energy
loss spectroscopy and the radiative and full electromagnetic local
density of states. ACS Photonics.

[10] Rossouw D., Couillard M., Vickery J., Kumacheva E., Botton G. A. (2011). Multipolar plasmonic
resonances in silver nanowire
antennas imaged with a subnanometer electron probe. Nano Lett..

[11] Qi R., Wang R., Li Y., Sun Y., Chen S., Han B., Li N., Zhang Q., Liu X., Yu D. (2019). Probing far-infrared surface phonon polaritons
in semiconductor nanostructures
at nanoscale. Nano Lett..

[12] Niedziółka-Jönsson J., Mackowski S. (2019). Plasmonics
with metallic nanowires. Materials.

[13] Zheng G., Mourdikoudis S., Zhang Z. (2020). Plasmonic metallic heteromeric nanostructures. Small.

[14] Wu Y., Li G., Camden J. P. (2018). Probing nanoparticle plasmons with
electron energy
loss spectroscopy. Chem. Rev..

[15] Borgh G., Bongiorno C., La Magna A., Mannino G., Shabani A., Patanè S., Adam J., Puglisi R. A. (2023). Plasmon resonances
in silicon nanowires: Geometry effects on the trade-off between dielectric
and metallic behaviour. Opt. Mater. Express.

[16] Borgh G., Bongiorno C., La Magna A., Mannino G., Patanè S., Adam J., Puglisi R. A. (2021). Surface plasmons in silicon nanowires. Adv. Photonics Res..

[17] Rafique R., La Magna A., Mio A. M., Patanè S., Adam J., Puglisi R. A. (2024). Plasmon response
in individual conical
silicon nanowires with different lengths. Photonics.

[18] Bosman M., Ye E., Tan S. F., Nijhuis C. A., Yang J. K. W., Marty R., Mlayah A., Arbouet A., Girard C., Han M.-Y. (2013). Surface
plasmon damping quantified with an electron nanoprobe. Sci. Rep..

[19] Rafique R., La Magna A., Mio A., Patanè S., Shrivastava A., Adam J., Puglisi R. A. (2024). Transversal
plasmon
resonance observed in tapered silicon nanowires. Int. Conf. Opt. MEMS Nanophotonics (OMN)..

[20] Liu J., Kan C., Cong B., Xu H., Ni Y., Li Y., Shi D. (2014). Plasmonic property
and stability of core–shell Au@SiO_2_ nanostructures. Plasmonics.

[21] Grady N. K., Halas N. J., Nordlander P. (2004). Influence
of dielectric function
properties on the optical response of plasmon resonant metallic nanoparticles. Chem. Phys. Lett..

[22] Chen W., Roca i Cabarrocas P. (2016). Insights into
gold-catalyzed plasma-assisted CVD growth
of silicon nanowires. Appl. Phys. Lett..

[23] Kolasinski K. W. (2006). Catalytic
growth of nanowires: Vapor–liquid–solid, vapor–solid–solid,
solution–liquid–solid and solid–liquid–solid
growth. Curr. Opin. Solid State Mater. Sci..

[24] Garozzo C. (2013). Competition between
uncatalyzed and catalyzed growth during the plasma
synthesis of Si nanowires and its role on their optical properties. J. Appl. Phys..

[25] Puglisi R. A., Bongiorno C., Caccamo S., Fazio E., Mannino G., Neri F., Scalese S., Spucches D., La Magna A. (2019). Chemical vapor
deposition growth of silicon nanowires with diameter smaller than
5 nm. ACS Omega.

[26] Shir D., Liu B. Z., Mohammad A. M., Lew K. K., Mohney S. E. (2006). Oxidation
of silicon nanowires. J. Vac. Sci. Technol.
B.

[27] Wagner R. S., Ellis W. C. (1964). Vapor–liquid–solid
mechanism of single
crystal growth. Appl. Phys. Lett..

[28] Putnam M. C., Filler M. A., Kayes B. M., Kelzenberg M. D., Guan Y., Lewis N. S., Eiler J. M., Atwater H. A. (2008). Secondary
ion mass spectrometry of vapor–liquid–solid grown, Au-catalyzed,
Si wires. Nano Lett..

[29] Moutanabbir O., Senz S., Zhang Z., Gösele U. (2009). Synthesis
of isotopically controlled metal-catalyzed silicon nanowires. Nano Today.

[30] Irrera A., Pecora E. F., Priolo F. (2009). Control of
growth mechanisms and
orientation in epitaxial Si nanowires grown by electron beam evaporation. Nanotechnology.

[31] Fontcuberta
Morral A., Arbiol J., Prades J. D., Cirera A., Morante J. R. (2007). Synthesis of silicon nanowires with wurtzite crystalline
structure by using standard chemical vapor deposition. Adv. Mater..

[32] Sivakov V., Andrä G., Himcinschi C., Gösele U., Zahn D. R. T., Christiansen S. H. (2006). Growth
peculiarities during vapor–liquid–solid
growth of silicon nanowhiskers by electron-beam evaporation. Appl. Phys. A: Mater. Sci. Process..

[33] Dhalluin F., Desré P. J., den Hertog M. I., Rouvière J.-L., Ferret P., Gentile P., Baron T. (2007). Critical condition
for growth of silicon nanowires. J. Appl. Phys..

[34] Zardo I., Conesa-Boj S., Estradé S., Yu L., Peiró F., Roca i Cabarrocas P., Morante J. R., Arbiol J., Fontcuberta
i Morral A. (2010). Growth study of indium-catalyzed silicon nanowires
by plasma enhanced chemical vapor deposition. Appl. Phys. A: Mater. Sci. Process..

[35] Ross F. M., Tersoff J., Reuter M. C. (2005). Sawtooth faceting
in silicon nanowires. Phys. Rev. Lett..

[36] Puglisi R. A., Bongiorno C., Borgh G., Fazio E., Garozzo C., Mannino G., Neri F., Pellegrino G., Scalese S., La Magna A. (2019). Study on the physico-chemical properties
of the Si nanowires surface. Nanomaterials.

[37] Reed B. W., Chen J. M., MacDonald N. C., Silcox J., Bertsch G. F. (1999). Fabrication
and STEM/EELS measurements of nanometer-scale silicon tips and filaments. Phys. Rev. B.

[38] Kikkawa J., Takeda S., Sato Y., Terauchi M. (2007). Enhanced direct interband
transitions in silicon nanowires studied by electron energy-loss spectroscopy. Phys. Rev. B.

[39] Adam T., Hashim U. (2013). COMSOL multiphysics
simulation in biomedical engineering. Adv. Mater.
Res..

